# A study on satisfaction with publicly financed health services in China

**DOI:** 10.1186/s12992-017-0292-y

**Published:** 2017-08-28

**Authors:** Shaoguo Zhai, Pei Wang, Anli Wang, Quanfang Dong, Jiaoli Cai, Peter C. Coyte

**Affiliations:** 1 School of Public Administration, Northwest University, 1 Xuefu Road, Chang’an District, Xi’an, Shaanxi 710127 China; 2 School of Politics and Administration, Xianyang Normal University, Wenlin Road, Xianyang, Shaanxi 712000 China; 3 School of Labor and Employment Relations, University of Illinois at Urbana-Champaign, 504 East Armory Avenue, Champaign, IL 61820 USA; 4 School of Public Policy and Administration, Xi’an Jiaotong University, 28 West Xian ning Road, Beilin District, Xi’an, Shaanxi 710049 China; 5 School of Economics, Wuhan University of Technology, 122 Luoshi Road, Wuhan, Hubei 430070 China; 6 Institute of Health Policy, Management and Evaluation, University of Toronto, Health Sciences Building, 155 College Street, Suite425, Toronto, ON M5T3M6 Canada

**Keywords:** Urban and rural residents, Publicly financed health services, Degree of satisfaction, China

## Abstract

**Background:**

With implementation of Chinese universal healthcare, the performance of urban and rural residents’ healthcare and the degree of satisfaction with publicly financed health services have become a hot issue in assessing health reforms in China. An evaluation model of health services in community and evaluation indexes of health-system performance have been put forward in related researches. This study examines variation in satisfaction with publicly financed health services among urban and rural residents in five Chinese cities and assesses their determinants.

**Methods:**

The data are derived from a survey of 1198 urban and rural residents from five nationally representative regions concerning their perceptions of satisfaction with China’s publicly financed health services. The respondents assessed their degree of satisfaction with publicly financed health services on a 5-point Likert scale. It is a kind of questionaire scale that features the answers for 1–5 points labeled very unsatisfied, unsatisfied, neither unsatisfied nor satisfied, satisfied and very satisfied linking to each factor or variable, where a score of 1 reflects the lowest degree of satisfaction and a score of 5 represents the highest degree. The logistic regression methods are used to identify the variables into its determining components.

**Results:**

The overall satisfaction degree representing satisfaction of all factors (variables) is 3.02, which is at the middle level of a 1–5 Likert scale, inferring respondents’ neutral attitude to publicly financed health services. According to the correlation test, the factors with characteristic root greater than 0.5 are chosen to take the factor analysis and 12 extracted factors can explain 77.97% of original 18 variables’ total variance. Regression analysis based on the survey data finds that health records, vaccinations, pediatric care, elder care, and mental health management are the main factors accounting for degree of satisfaction with publicly financed health services for both urban and rural residents.

**Conclusions:**

What can be done to increase the degree of satisfaction with health services needs to be considered based on our findings. Regression analysis based on the survey data finds that health records, vaccinations, pediatric care, elder care, and mental health management are the main factors accounting for degree of satisfaction with publicly financed health services for both urban and rural residents. Therefore, with improvements in health records, timely vaccination, elder care for women or elder, pediatric care and major psychosis management, degree of satisfaction with publicly financed health services are likely to grow.

## Background

Chinese healthcare reforms are designed to combine four types of health services, including public health (public financed health services), health insurance, health services, and drug supply for both urban and rural residents in order to form one integrated basic health care system (the Central People’s Government of the People’s Republic of China, 2016). Overall satisfaction with health services is generally related to the quality of the services received or to be received. Concerns about variations in access to quality publicly financed health care services for urban and rural residents are all contemporary public policy concerns in China. With implementation of universal healthcare, the performance of urban and rural residents’ healthcare and the degree of satisfaction with publicly financed health services have become a hot issue in assessing health reforms in China.

Chinese public health policy is more and more closely in accordance with the developing trend of international public health services. Satisfaction with publicly financed health services is one important indicator to measure health system performance and its evaluation index system is the main issue focused by previous researchers. Thomas [1] classified healthcare into three types (primary care, secondary care and tertiary care) from low level to high level, according to healthcare activities, specialization and severity. Zhang [2] classified healthcare into three categories: public healthcare, quasi public healthcare and private properties of healthcare services. Although some scholars believed that contracting for the delivery of health services could be very effective and that kind of improvements could be rapid [3, 4], the priority arrangement in healthcare, which was of growing importance, promoted the combination of constrained resources and the increasing demands of healthcare [5]. International studies of health services satisfaction evaluation started earlier, and the evaluation system is still improving. Shackley and Donaldson [6] addressed that the willingness to pay for publicly-financed healthcare could reveal the residents’ satisfaction to some extent. At present, relatively mature international evaluation model includes four models: Donabedian evaluation model, which focuses on “the organization structure, the health resources, work and the result”; Italy’s Piedmont evaluation model, which emphasizes on “behavior index, equality index, productivity index and life quality index”; Sackett services object model and Parker’s system evaluation model [7]. Arnold Leving [8] suggested the Delphi expert consultation is the effective way to establish index system with a program selection on purpose.

Chinese scholars have begun a preliminary exploration of related evaluation contents and index of healthcare based on health system performance assessment framework suggested by WHO. Generally, China’ s evaluation model for community public health services can be divided into two models,“support-process-effect” model and “devotion-service-efficiency” model, and both of them have their own specific support index. In terms of index system, Liang [9] established a set of evaluation index system of China’s urban community health services with Delphi expert consultation for two rounds of inquiry by gathering experts from domains of general medicine, clinical medicine, health education and health economics. Cui [10] also used this kind of method to summarize the indexes from aspects of health input, public health services items, health services management, services implementation effect and services charge and performed the results from experts in SPSS, getting the arithmetic average, weighted average, variation coefficient and cooperation index of the experts’ suggestions in questionnaire and Ni [11] assumed the exercise and fitness, health supervision and coordination services, elder healthcare and health education are main indexes whose weight coefficient are top high by the Delphi method. Chen [12] calculated the weight of indexes with fuzzy comprehensive evaluation, concluding that the highest weight coefficient is the aspect of service items including vaccination, children and women healthcare, infectious diseases and health emergence, health education and major psychosis management. Yu et al. [13] carried out the dimensionless method targeting on the features of evaluation system, getting the comprehensive performance index of public health services and finally obtained 4 main indexes: health care spending, reimbursement range, monthly health situation and physical examination times. Xu and Deng [14] analyzed factors summarized from previous scholars employing the correlative and factor analysis and got changes in health situation, resident health record and health supervision were the main factors affecting the satisfaction of public health services. Chen and Li [15] and Tian [16] employed the RSR method AHP method to confirm that the index system consists of annal hospitalization, chronic diseases management, major psychosis management, etc. Liang [9] adapted the Crowns Bach coefficient method and the principal component factor analysis to evaluate the validity and reliability of community health services model’s index system. In terms of actual investigation and study, Zhou et al. [17] analyzed community health services performance from the level of hospital services and community health services for the first time and measured the response level of community health services in Shenzhen using one-way ANOVA and Fuzzy math assessment. Huang et al. [18] conducted a research of Kwangtung in China on the satisfaction of public health, resulting to the idea that 70% to 85% of respondents are satisfied with public health management of the society. Zhang [19] also verified the result that health information management, health education, resident health record and children, women or elder healthcare are the essential indexes with Delphi method when all of experts’ suggestions tending to uniform. The methods mentioned above are employed to select and screen factors or indexes in the public health performance evaluation system. We chose the selected indexes according to mature and former scholars’contribution to conduct second confirmation with a new model like the multiple linear regression model and logistic regression model, getting more exact and accurate factors affecting the satisfaction. Before that, we used Likert scale to investigate the level of satisfaction, getting the attitude to public health services of 1198 urban and rural residents in five cities (Zhenjiang, Dongguan, Chengdu, Shenmu, Yinchuan) from the perspective of the demand side of health services, which is creative compared with the former researches from the aspects of services providers. The project also analyzed the factors of public health services based on the empirical analysis in order to explore how to improve the satisfaction with public health services among urban and rural residents.

This paper is organized in the following manner. In Methods, the methods are presented. Descriptive statistics analysis reports descriptive results on satisfaction by urban and rural residents with publicly financed health services. Factor and multivariate regression analyses that account for variations in satisfaction are presented in Results and Discussions, respectively. Conclusions and Limitations present the conclusions and limitations.

## Methods

### Data source

An interview-based questionnaire was designed to assess perceived satisfaction with public health services for a random sample of 1250 urban and rural residents in five Chinese regions: Zhenjiang, Dongguan, Chengdu, Shenmu, and Yinchuan. We chose these five cities by deep discussions with scholars in healthcare field. These five regions, which include both urban and rural areas, are reflective of different levels of socioeconomic development in China and they were selected to be broadly representative of both high-income and low-income populations. Of the 1250 surveys distributed, there were 1220 respondents, but only 1198 were complete and usable, yielding an effective participation rate of 95.8%. The main reason for non-response was an inability by study subjects to understand and accurately report answers to the survey questions. We declare that we have no conflict of interest in procedure of the survey. All procedures performed in studies involving human participants were in accordance with the ethical comparable standards of the institutional or national research committee. Informed consent was obtained from all individual participants included in the study.

### Definition of variables, reliability and validity

The respondents assessed their degree of satisfaction with publicly financed health services on a 5-point Likert scale, where a score of 1 reflects the lowest degree of satisfaction and a score of 5 represents the highest degree of satisfaction (Table 1). The reliability coefficient is 0.974, which suggests that reliability of this questionnaire is high. Content validity refers to the number of topics represented by the scale, and the correlation between each score and the total score can be used as an indicator of the validity of the questionnaire. The correlation coefficients for each category score and the total score are greater than 0.70 and are statistically significant, which means the overall questionnaire content is valid.

**Table 1:** Basic public health service satisfaction average table

Average	Total	Rural	Urban
Resident health record	3.04(0.894)	3.06(0.801)	2.81(0.909)
Health education	3.11(0.869)	3.16(0.819)	2.93(0.939)
Vaccination	3.24(0.844)	3.28(0.811)	3.13(0.935)
Infectious diseases and health emergence	3.06(0.878)	3.12(0.894)	2.94(0.965)
Children healthcare	3.10(0.906)	3.17(0.904)	2.97(0.941)
Women healthcare	3.07(0.893)	3.13(0.888)	2.92(0.882)
Elderly healthcare	2.94(0.964)	3.01(0.919)	2.77(1.042)
Chronic diseases management	2.89(0.866)	2.98(0.815)	2.76(0.905)
Major psychosis management	2.90(0.878)	2.96(0.800)	2.83(0.923)
Health supervision and coordination service	2.82(0.954)	2.88(0.891)	2.66(0.928)
Total Satisfaction	3.00(0.779)	3.06(0.690)	2.88(0.827)

Standard errors in parentheses

### Conceptual model

We firstly plan to employ multiple linear regression to get the accurate factors affecting the satisfaction with publicly financed health services based on the factor analysis which can extract the main components from all of variables. So we would use the following conceptual models of factor analysis (1) and multiple linear regression (2). Then we would employ the ordinal logistic regression model (3) to confirm the result. Before we undertake factor analysis to extract main components for use of multiple linear regression, we need to screen the variables and carry forward the correlation analysis in order to get the cumulative variance contribution ratio of the factors.

1 $$ Fj=u1 jX1+u2 jX2+u3 jX3+\dots + upjXp\left(j=1,2,\dots, m\right) $$

2 $$ Y=a+b1X1+b2X2+b3X3+\dots + bkXk+e $$

3 $$ \log it\left(\pi 1\right)=-\left(-\alpha 1+\beta 1X1+\dots +\beta mXm\right) $$

For the correlation analysis part, we initially review ten potential determinants of satisfaction and then conduct a correlation test to analyze them. They all passed the correlation test. Detailed data can be seen in Table 2. For selecting variable, we change Ordinal Scale into Interval Scale and observe variable correlation coefficient matrix. If the correlation coefficient between a variable and most of the other variables are small (less than 0.100), the variable will be deleted. We use KMO inspection to determine whether the selected variables are suitable for factor analysis or not so Table 3 shows that the KMO value of variables is 0.895, which is more than 0.7, meaning that the variables we selected are suitable for factor analysis. In addition, *p*-values are less than 0.001 and the original variables are suitable for factor analysis. At last, we omitted the variable with more missing value.

**Table 2:** Spearman coefficient of basic public health service variation

Resident health record	Correlation Coefficient	−.606^**^	Children healthcare	Correlation Coefficient	−.635^*^	Chronic diseases management	Correlation Coefficient	−.598^**^
	Sig.(2-tailed)	.000		Sig.(2-tailed)	.000		Sig.(2-tailed)	.000
Health education	Correlation Coefficient	−.614^**^	Women healthcare	Correlation Coefficient	−.608^**^	Health supervision and coordination	Correlation Coefficient	−.547^**^
	Sig.(2-tailed)	.000		Sig.(2-tailed)	.000		Sig.(2-tailed)	.000
Vaccination	Correlation Coefficient	−.579^*^	Elderly healthcare	Correlation Coefficient	−.635^**^			
	Sig.(2-tailed)	.000		Sig.(2-tailed)	.000			
Infectious diseases and health	Correlation Coefficient	−.605^**^	Chronic diseases management	Correlation Coefficient	−.603^**^			
	Sig.(2-tailed)	.000		Sig.(2-tailed)	.000			

(** means ‘*P* value<=0.01’, * means ‘*P* value<=0.05’ )

**Table 3:** KMO&Bartlett’s test of sphericity

KMO measure of sampling adequacy		.895
Bentley’s sphericity test	Approx. Chi-Square	574.669
	Df	253
	Sig.	.000

After screening procedure above, we finally left 18 variables to get ready to the factor analysis. They are clearly presented in the Table 4. We can also learn about the common factor variance of 18 variables based on the characteristic root less than 0.5. And we definitely get the cumulated variance contribution ratio of the factors in Table 5, implying that the root value of first factor’s characteristics is 6.591 that explains 28.66% of original 18 variables’ total variance. The root value of second factor’s characteristics is 1.548, which explains 6.73% of original 18 variables’ total variance, the next 16 variable can be seen by parity of reasoning. As concluded, 12 extracted factors can explain 77.97% of original 18 variables’ total variance so we choose these 12 factors into our next analysis framework. Overall, the loss of information of original variables is small and the effect of the factor analysis is good.

**Table 4:** The initial solution of factors (characteristic root value is 0.5)

Factor	Initiation	Extraction	Factor	Initiation	Extraction
Health care spending range	1.000	.960	Vaccination	1.000	.627
Two-week prevalence rate	1.000	.922	Infectious diseases and health emergence	1.000	.626
Reimbursement range	1.000	.857	Children healthcare	1.000	.749
Monthly health situation	1.000	.881	Women healthcare	1.000	.730
Annual hospitalization	1.000	.760	Elderly healthcare	1.000	.655
Physical examination times	1.000	.904	Chronic diseases management	1.000	.669
Exercise & Fitness	1.000	.932	Major psychosis management	1.000	.708
Changes in health situation	1.000	.937	Health supervision and coordination service	1.000	.691
Resident health record	1.000	.701			
Health education	1.000	.654

**Table 5:** Total variance explained of factors

Component	Total variance explained								
	Initial eigenvalues			Extraction sums of squared loadings			Rotation sums of squared loadings		
	Total	% of Variance	Cumulative %	Total	% of Variance	Cumulative %	Total	% of Variance	Cumulative %
1	6.591	28.658	28.658	6.591	28.658	28.658	2.294	9.972	9.972
2	1.548	6.729	35.388	1.548	6.729	35.388	2.268	9.863	19.835
3	1.428	6.210	41.597	1.428	6.210	41.597	2.176	9.460	29.295
4	1.307	5.682	47.280	1.307	5.682	47.280	1.978	8.601	37.896
5	1.121	4.873	52.153	1.121	4.873	52.153	1.796	7.810	45.706
6	.976	4.243	56.396	.976	4.243	56.396	1.184	5.150	50.856
7	.926	4.025	60.421	.926	4.025	60.421	1.070	4.652	55.508
8	.919	3.994	64.415	.919	3.994	64.415	1.054	4.584	60.092
9	.847	3.682	68.096	.847	3.682	68.096	1.044	4.540	64.631
10	.795	3.456	71.553	.795	3.456	71.553	1.029	4.474	69.105
11	.767	3.334	74.887	.767	3.334	74.887	1.021	4.440	73.545
12	.710	3.085	77.972	.710	3.085	77.972	1.018	4.427	77.972
13	.671	2.918	80.890						
14	.634	2.758	83.647						
15	.572	2.488	86.135						
16	.565	2.458	88.594						
17	.478	2.077	90.671						
18	.452	1.966	92.637						
19	.417	1.814	94.451						
20	.400	1.741	96.192						
21	.372	1.618	97.810						
22	.306	1.332	99.142						
23	.197	.858	100.000						

Extraction Method: Principal Component Analysis

## Descriptive statistics analysis

Through descriptive statistics, we may get a macro view of our sample’s information and respondents’ basic information such as their age, educational background, hospitalization rate, frequency of taking exercise and monthly health condition shown in Table 6. As to satisfaction with publicly financed health services displayed in Table 1, we can understand overall satisfaction with publicly financed health services, the different scores and their specific characteristics among different people and regions, describing public health services satisfaction by the use of Likert scale so we interpret the mean of each indexes of satisfaction which indicated the general level of public health satisfaction. Overall satisfaction with basic publicly financed health services is 3.00, which is in the mid-range on the five-point scale and is reflective of neither dissatisfaction nor satisfaction with services. Among these scores, satisfaction score for health supervision and coordination services is 2.82, lower than other scores, while satisfaction with vaccinations is at the other end of the spectrum with a score of 3.24. From the perspective of different people, rural residents report the highest overall satisfaction score, with a score 3.00; while urban residents have the lowest satisfaction score which is 2.88. From an overall perspective, satisfaction scores for publicly financed health services in five typical areas of China (Zhenjiang, Guangdong, Chengdu, Shenmu, Yinchuan) are all around 3. We can conclude that satisfaction with health services for urban and rural residents is neutral. Detailed data can be seen in Table 1.

**Table 6:** Descriptive characteristics of survey respondents

Statistical index		Proportion (%)
Regions	Zhenjiang	19.3
	Dongguan	16.8
	Chengdu	24.8
	Shenmu	21.1
	Yinchuan	18.0
Age Distribution	20 and Below	3.9
	21–40	53.3
	41–60	29.4
	61 and Above	12.2
	Not Available	1.1
Gender Proportion	Male	51.5
Groups	Workers	37.5
	Urban Residents	28.0
	Peasants	34.5
Occupational Distribution	Civil Servants and Staff of Public Institutions	20.4
	Staff of Foreign-Funded and Private Enterprises	14.5
	Staff without Fixed Employment	59.3
	Retirees	6.8
Educational Background	Primary School and Below	15.5
	Middle School	46.4
	Junior College and Above	37.8
Household Registration Types	Local	62.4
	Non-Local	14.4
	Not Available	23.3
One-year Hospitalization Rate	Yes	10.2
	No	87.1
	Not Available	2.7
Take Exercise	Frequently	27.8
	Sometimes	52.6
	Never	19.2
	Not Available	0.4
Monthly Health Condition	Very Good	16.4
	Good	36.6
	Average	38.8
	Poor	6.1
	Very Poor	0.8
	Not Available	1.3
Incidence of Chronic Disease	Yes	14.9
	No	85.1
Two-week Incidence	Yes	8.5
	No	88.5
	Not Available	3.0

## Results

### Factor explanation for factor analysis

We will get 12 new components extracting from all 18 factors shown in Table 7. According to the table, chronic diseases management, major psychosis management and health supervision, and coordination service have higher load on factor F1. The first factor F1 mainly explains these three variables, so the factor F1 can be regarded as a disease management supervision factor. Child healthcare, women healthcare and elderly healthcare have higher load on factor F2, so the second factor F2 can be regarded as healthcare factor. Resident health record, health education, vaccination, infectious diseases and health emergence have higher load on factor F3, so factor F3 can be referred to as health intervention and prevention factors. Reimbursement range and annual hospitalization have higher load on factor F6, so factor F6 can be regarded as hospital and reimbursement factor. Physical examination times has higher load on factor F7, which is regarded as physical factor. Two-week prevalence rate has higher load on factor F8, which is regarded as Two-week prevalence rate factor. Monthly health situation has higher load on factor F9, which is regarded as health factor. Healthcare spending range has higher load on factor F10, which is regarded as the factor of healthcare spending. Changes in health situation has higher load on factor F11, which is regarded as the health change factor; exercise and fitness has higher load on factor F12, which is regarded as exercise health factor. The component factor 4 and 5 cannot explain variables well so we excluded them. Therefore, we screen 10 new components for the next analysis by putting them into the multiple linear as independent variables when the satisfaction with the public health served as dependent variable. We can learn how those 10 variables are used in the part 4.2.

**Table 7:** After rotating factor loading table

	Component											
Factor	1	2	3	4	5	6	7	8	9	10	11	12
Health care spending range	.005	−.035	−.017	.030	.048	.019	.077	−.033	.067	.969	.017	−.061
Two-week prevalence rate	−.042	−.066	.021	.071	.018	.041	.043	.947	−.096	−.034	.006	.022
Reimbursement range	.002	.080	−.150	−.017	.052	.886	−.114	.136	−.037	.031	−.010	−.081
Monthly health situation	.085	.082	.003	−.039	−.060	.032	.039	−.110	.903	.071	.159	.041
Annual hospitalization	.075	.239	−.179	−.148	.083	−.607	−.367	.284	−.185	.022	−.047	−.125
Physical examination times	−.013	.013	−.108	−.005	.007	−.018	.928	.054	.025	.082	−.017	−.140
Exercise & Fitness	.098	.047	.004	−.087	−.009	−.032	−.134	.019	.044	−.062	.106	.936
Changes in health situation	.008	.070	.030	−.030	−.073	.008	−.013	.006	.152	.016	.944	.104
Resident health record	.331	.098	.675	−.226	−.078	−.085	−.048	.000	.052	−.200	.096	−.089
Health education	.258	.180	.677	−.251	−.104	−.043	−.088	.045	.015	−.005	.081	−.060
Vaccination	.120	.424	.595	−.012	−.218	.034	−.103	.035	.019	.069	−.073	.087
Infectious diseases and health emergence	.243	.373	.596	−.091	−.104	−.032	.067	−.081	−.084	.112	−.071	.128
Children healthcare	.093	.763	.328	−.139	−.132	−.056	.047	−.008	.061	−.012	−.023	.063
Women healthcare	.261	.766	.151	−.162	−.077	.002	−.017	−.046	.035	−.043	.108	.043
Elderly healthcare	.408	.630	.164	−.186	−.088	.044	−.050	−.038	.045	−.036	.062	−.095
Chronic diseases management	.699	.268	.245	−.122	−.076	.006	.032	−.102	−.034	−.006	.014	.120
Major psychosis management	.755	.211	.236	−.123	−.131	−.015	−.017	−.032	.019	−.006	.057	.024
Health supervision and coordination service	.749	.120	.160	−.174	−.183	−.033	−.035	.066	.120	.022	−.061	.028

### Results for multiple linear regression model

According to the factor extraction by the factor analysis we obtain 10 new and main components mentioned above. By using this model, 10 factors are used as independent variables to account for variations in reported satisfaction with publicly financed health services.

R squared of the model is 0.994 which suggests that the model fits the data well. Table 8 shows that the *p*-value is 0.000, which is smaller than 0.01 and means the regression equation is highly significant. Ten factors’ score models are shown Table 9. It is clear that some of 10 components are significant (*p* < 0. 000), but exercise health factor, healthcare spending and Two-week prevalence rate factor are not significant with the *p*-value more than 0.01 so that only seven components are left in the final regression equation. So we get the regression equation according the coefficients as followed:

$$ Y=0.789-0.097{F}_1-0.120{F}_2+0.249{F}_3-0.026{F}_6+0.070{F}_7-0.020{F}_9+0.028{F}_{11} $$

**Table 8:** Analysis of variance table

Model		Sum of Squares	Df	Mean Square	F	Sig.
1	Regression	8.186	12	.682	935.340	.000
	Residual	.046	63	.001		
	Total	8.232	75

**Table 9:** The regression equation coefficient table

Model	Unstandardized Coefficients		Standardized Coefficients	t	Sig.	Correlations		
	B	Std. Error	Beta			Zero-order	Partial	Part
(Constant)	.789	.003		254.855	.000			
F12	−.001	.003	−.004	−.464	.644	−.004	−.058	−.004
F11	.028	.003	.083	8.819	.000	.083	.743	.083
F10	.005	.003	.016	1.749	.085	.016	.215	.016
F9	−.020	.003	−.060	−6.347	.000	−.060	−.625	−.060
F8	−.001	.003	−.003	−.354	.725	−.003	−.045	−.003
F7	.070	.003	.212	22.514	.000	.212	.943	.212
F6	−.026	.003	−.078	−8.265	.000	−.078	−.721	−.078
F5	−.031	.003	−.094	−10.028	.000	−.094	−.784	−.094
F4	.124	.003	.374	39.709	.000	.374	.981	.374
F3	.249	.003	.753	79.986	.000	.753	.995	.753
F2	−.120	.003	−.363	−38.583	.000	−.363	−.979	−.363
F1	−.097	.003	−.292	−31.041	.000	−.292	−.969	−.292

According to regression equation, satisfaction with publicly financed health services depends on seven main factors, they are disease management supervision factor (F1), healthcare factor (F2), health intervention and prevention factors (F3), hospital and reimbursement factor (F6), physical factor (F7), health factor (F9), and health change factor (F11). These factors are also related to the satisfaction reported by urban and rural residents with different coefficient values. F1, F2 and F6 exert negative effects on it, while F9, F3, F7 and F11 are positively correlated.

### Results for the confirmation of logistic regression model

After we get the final factors affecting satisfaction with public health services by the multiple linear regression, we next employ the ordinal logistic regression model to confirm that. We also regard 10 components mentioned above as independent variables with the satisfaction with publicly financed health services served as dependent variable. It is explicitly reflected in Table 10.

**Table 10:** Logistic regression analysis of satisfaction of public health services of urban and rural residents (Sig. coefficient 0.05)

	B	S.E.	Wald	df	Sig.	Exp(B)	95% C.I. for EXP(B)	
							Lower	Upper
Infectious diseases and health emergence	−.329	.137	5.818	1	.016	.719	.550	.940
Chronic diseases management	.329	.148	4.952	1	.026	1.390	1.040	1.858
Resident health record	.782	.221	12.524	1	.000	2.186	1.418	3.371
Vaccination	.964	.218	19.514	1	.000	2.621	1.709	4.020
Children healthcare	1.212	.228	28.208	1	.000	3.360	2.148	5.256
Women healthcare	.493	.236	4.361	1	.037	1.637	1.031	2.601
Elderly healthcare	.514	.233	4.869	1	.027	1.673	1.059	2.642
Major psychosis management	−.672	.262	6.568	1	.010	.511	.306	.854
Constant	3.882	1.483	6.849	1	.009	48.533		

## Discussions

In order to comprehensively analyze the main determinants of satisfaction reported by urban and rural residents, we create a binary satisfaction variable that is 1.0 when reported as neutral or better satisfaction (ie they reported being “totally satisfied”, “satisfied” or “neutral”) and 0 otherwise. We choose binary variable as independent variable because it was often used in reliable literatures and it was easier to understand the result when using binary variable. To assess the determinants of satisfaction, we use the same set of variables that were previously reported to be significant, as shown in Table 10. These results imply that infectious diseases and health emergence, chronic diseases management, resident heath record, vaccination, children healthcare, women healthcare, elderly healthcare and major psychosis management whose *P* value are less than 0.05 based on the 95% confidence interval are the main factors affecting public health satisfaction. The odds ratio in this retrospective study which represented the relative risk reduction in prospective study can be seen from the Exp(B). Take chronic diseases management for example, the odds ratio of it is 1.390, so this kind of management contributes 0.39(1.39–1) to the satisfaction with public health services. According to the significance level, resident health record, vaccination, child healthcare, elder healthcare, major psychosis management have significant correlation with satisfaction. Therefore, improving health records, timely vaccination, elder care for women or elder, pediatric care and major psychosis management will exert conducive influence on satisfaction with publicly financed health services.

We surmise that introducing specific public health policy aiming at special groups, detailed and informative public health management, public healthcare publicity and accessible public health services is effective measurement that can be taken smoothly by public sectors. We also learn what can be done to improve satisfaction with publicly financed health services from previous researches literatures. Liang [9] suggested that government should take the burden of public health and intensify its role-playing with the forced supervision and management. Cui [10] hold ideas that the barren and poverty-stricken areas need to be input in public health. Ni [11] concluded that general practitioner training should be carried out in order to strengthen the guidance of medical care. Huang et al. [18] revealed in his researches in Kwangtung that policy related to public health need to adept to the different groups according to different social economics status. Our research summarize following conclusions referring to statistical analysis.

### Specific public health policy emphasizing on special groups

Public health services system is not established and improved according to economic conditions and demographic characteristics in different regions. Research find that people’s health knowledge of disease control and prevention consciousness, rescue consciousness are weak, which require the government, health care, hospitals, health posts, clinic propaganda strengthening to raise the level of health services. It is important to emphasize on the importance of preventive care to form the integration of prevention, treatment, rehabilitation and health promotion reimbursement mechanism, guiding and motivating health services providers to form “preventive care and health promotion” services mode. Public sectors need to put medical insurance funds into basic health care and health promotion activities, which have lower input and higher output to promote the health level.

### Detailed and informative public health management

Management is not detailed and informative enough to manage disease, advocate health education, execute the local common disease supervision and provide better advice, doctor, medical treatment, hospitalization, rehabilitation exercise and healthy environment. On the basis of full coverage, there should be a focus on the improvement of reimbursement ratio, implementation of fine management and information management for common diseases or multiple incidence. It should be realized to cover the whole national health management and services through information management which is defined by universal health care services including the maternal and infant health care, birth records, child care (free vaccination, physical examination), adult health care (health, family planning guidance, department of gynecology, community rehabilitation, health education and promotion), the elder healthcare (old man check-up, chronic disease management, health education and promotion, health assessment, the old man follow-up, domestic sickbed), to hospice care, which is a whole health services system from birth to death of holistic.

### Public healthcare publicity

Healthcare prevention propaganda and different groups of medical and healthcare specifications are not implemented to enhance people’s health and health self-test practice, to vigorously promote healthy exercise, and to improve urban and rural residents’ health level. Due to the reasons of lacking of health care awareness, economic limitation and uncovered reimbursement procedures or conditions, many people don’t seek medical advice even if they need or should. People do not have enough attention on their health with the healthy concept incompletely implemented. Thus, invigorating and stimulating the health and prevention health care consciousness is essential. According to the requirements of the health management and the cost of chronic disease intervention effect, infrastructure health services institutions to provide basic medical and health care services should be guided and standardized, which emphasizes the prevention of chronic diseases prevention and health promotion. The public sectors also need to provide comprehensive health management which is convenient, available, technology-appropriate, and cost-reasonable to urban and rural residents.

### Accessible public health services

Dual structure of urban and rural is not clearly divided, a fair and sound health security system consisted with constructed and basic health services is not provided. The survey results reflect that different regions and different groups all agree that the disparity and inequality of basic public health services exits between urban and rural. There is a consensus for the difference between the urban and rural health security system in our country residents, which is confirmed with academic research. The reason of the inequality is related to government fiscal imbalance, unequal distribution of public health resources, and regional differences in economic development. In the process of health security system construction, government should balance interests between different groups of people and different regions, and satisfy or dig the healthcare needs of residents. Health management platform for urban and rural residents should be established and carried out to provide basic health services ensuring the fairness and accessibility of basic health services.

## Conclusions

What can be done to increase the degree of satisfaction with health services needs to be considered based on our findings. Regression analysis based on the survey data finds that health records, vaccinations, pediatric care, elder care, and mental health management are the main factors accounting for degree of satisfaction with publicly financed health services for both urban and rural residents. Therefore, with improvements in health records, timely vaccination, elder care for women or elder, pediatric care and major psychosis management, degree of satisfaction with publicly financed health services are likely to grow.

## Limitations

There are several limitations in this study. First up, the random selection only covers 1250 urban and rural residents in five Chinese regions. We hope to expand the scope of the survey and increase sample size to enhance the conclusion we have got from this survey in the future although our sample is representative enough. Moreover, some results of the sample are missing or distorted in this survey because of some uncontrollable and subjective reasons (for example, the limitation of the resident’s culture, especially in some undeveloped areas) affecting the results. What’s more, some omitted factors leading to inaccurate estimates probably make the impact factors incomprehensible although we have controlled variables as much as possible. Exceptionally, the research methodology is simplified among other models which lead the similar results, we just use the oridinal logistic regression model to confirm the result, but we didn’t list all of them one by one, it would be better if it can include other models to confirm the results and show reasons why some models can not be employed. In the future, the study will focus on collecting more data from other reign of China and make a comparison between different models for this motif. At last, we did not took subjective factors into account, this is what we will do in further researches.

## Abbreviations

AHP: Analytic Hierarchy Process
ANOVA: analysis of variance
KMO: Kaiser-Meyer-Olkin
RSR: Rank Sum Rank
WHO: World Health Organization
